# Ferroptosis: A New Strategy for Cancer Therapy

**DOI:** 10.3389/fonc.2022.830561

**Published:** 2022-02-16

**Authors:** Yu Chen, Zhihua Fan, Shen Hu, Chengchao Lu, Yi Xiang, Shuzhi Liao

**Affiliations:** Department of Pediatric Surgery, Guangdong Women and Children Hospital, Guangzhou, China; ^2^ Department of Dermatology, Xiangya Hospital, Central South University, Changsha, China; ^3^ Department of Obstetrics, Second Affiliated Hospital, Zhejiang University School of Medicine, Hangzhou, China

**Keywords:** ferroptosis, cancer, iron, programmed cell death, clinical application

## Abstract

Ferroptosis is a newly discovered form of iron-dependent cell death, which is different from other death forms. The main characteristics of ferroptosis are: (1) Amino acid metabolism. (2) Iron metabolism; (3) Lipid metabolism and Reactive oxygen species (ROS). Ferroptosis is related to the occurrence and development of a variety of cancers, especially in the drug resistance. This article reviews the research progress of iron death in tumors, and provides a theoretical reference for its further research and clinical application.

## Introduction

Cell death is essential for maintaining the body’s normal development, homeostasis, and preventing hyperproliferative diseases (such as cancer). The process of regulatory cell death is regulated by specialized molecular mechanisms, so it can be regulated by specific pharmacological methods or genetic intervention (1). Ferroptosis is a newly discovered programmed cell death event. Ferroptosis is different from death forms such as apoptosis, necrosis, and autophagy in terms of morphology, biochemistry, and gene regulation. It does not require energy consumption, is not inhibited by apoptosis inhibitors, and has no intracellular calcium overload (2). Ferroptosis is mainly marked by a significant increase in cytoplasmic iron and lipid ROS, a decrease in mitochondrial volume, and an increase in the thickness of the bilayer membrane (3). In clinical treatment, the multi-drug resistance of cancer cells is one of the important reasons for treatment failure (4). The discovery of ferroptosis provides new ideas for the treatment of cancer and the solution of drug resistance.

## The Discovery of Ferroptosis

**Figure 1:**
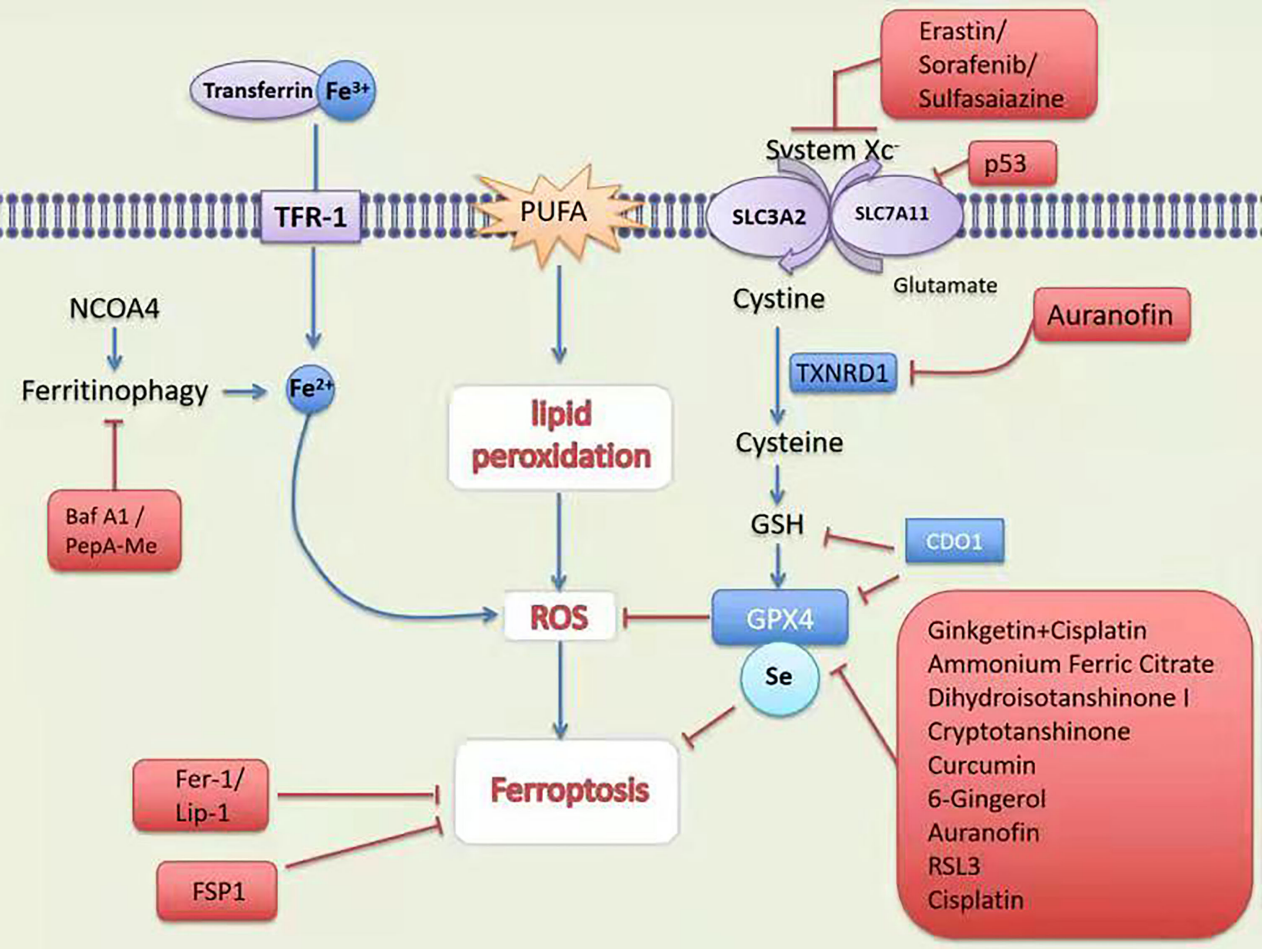
Mechanism and treatment of ferroptosis. TFR-1, transferrin receptor 1; PUFA, polyunsaturated fatty acids; SCARA5, scavenger receptor class A member 5; SLC1A5, solute carrier family member 1 member A5; System Xc-,contribution of the cystine-glutamate antiporter; NCOA4; Nuclear receptor coactivator 4; GSH, glutathione; Fer-1, ferrostatin-1;Lip-1, lipoxstatin-1; FSP1, ferroptosis suppressor protein 1; GPX4, glutathione peroxidase 4; p53, tumor suppressor protein 53; TXNRD1, thioredoxin reductase 1; CDO, cysteine dioxygenase 1; RSL3, RAS-selective lethal 3.

**Figure 2:**
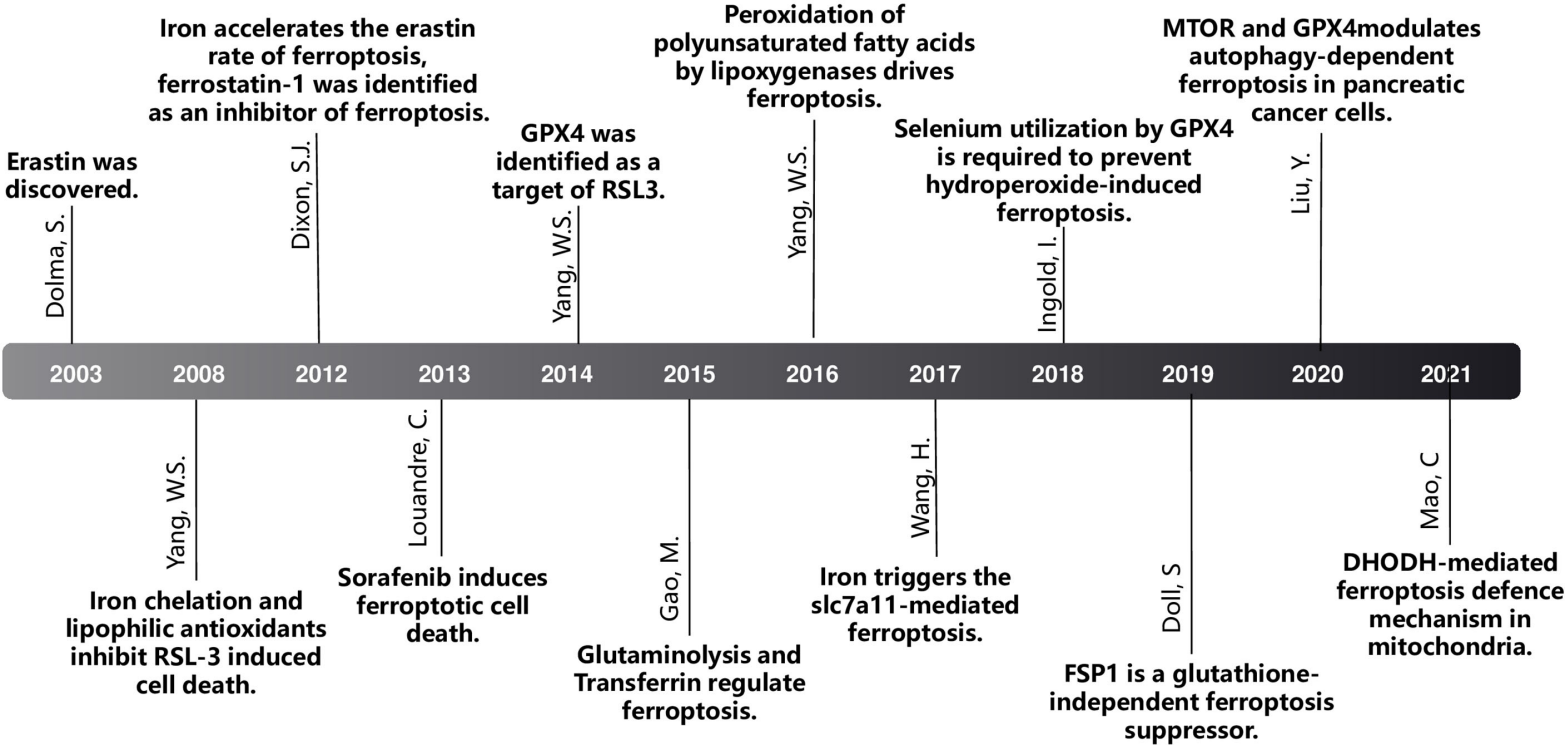
The milestone of ferroptosis.

DOLMA et al. (4) discovered an anti-tumor drug in 2003, which can induce cell death without causing changes in nuclear morphology, DNA fragmentation and caspase3 activation, and caspase inhibitors cannot reverse this process. Yang et al. (5) discovered the compounds RSL3 and RSL5 that cause this new type of cell death and found that iron chelating agents can inhibit this type of death. In 2012, Dixon et al. (6) were studying tumor cells with RAS mutations. At the time, for the first time, a non-apoptotic iron-dependent cell death method caused by Erastin was defined as ferroptosis (**Figure 1**). In the following years, several studies confirmed that the reduction of intracellular cysteine ​content and the massive consumption of GSH played a key role in inducing cell death and proved that lipophilic antioxidants and iron chelators can inhibit such death way, these are the main features of ferroptosis (**Figure 2**).

## The Process of Ferroptosis

The main characteristics of ferroptosis are: (1) Amino acid metabolism. (2) Iron metabolism; (3) Lipid metabolism and ROS

### Amino Acid Metabolism

Amino acid metabolism is an important part of the organism’s metabolic circuit. Abnormal amino acid metabolism is closely related to ferroptosis (7). Glutathione is a tripeptide compound composed of 3 amino acids glutamic acid, cysteine ​​and glycine. It is important in the body Antioxidant and free radical scavenger (8). Glutathione can combine with free radicals, heavy metals, etc., to convert harmful poisons in the body into harmless substances to be excreted from the body (9). The structure of glutathione contains a lively sulfhydryl group-SH, which is easily oxidatively dehydrogenated. This specific structure makes it the first line of defense for the elimination of free radicals in the body (10). System Xc^-^ plays an important role in maintaining the balance and distribution of glutathione. Xc^-^ depends on sodium ion and is composed of light chain subunits and heavy chain subunits (11). Xc^-^can transfer glutamic acid in the cell to the outside of the cell, and at the same time transfer the extracellular cystine into the cell and regulate the synthesis of glutathione by affecting the level of extracellular glutamate. Studies have found that Xc^-^ knockout mice have significantly lower levels of glutamate outside the brain cells and have a milder neurotoxic response to drugs (12).

Erastin is one of the small molecules found in chemical screening that can induce ferroptosis in oncogenic RAS mutant cell lines. In the process of Erastin-induced ferroptosis, System Xc^-^ is the most important mechanism (13). The transporter is composed of SLC7A11 and SLC3A2. It can take up extracellular cystine into the cell at a ratio of 1:1 and is quickly reduced to cysteine ​to participate in the synthesis of GSH (14). GPX4 is an antioxidant enzyme containing selenoprotein in cells, which is mainly synthesized in the proximal tubules of the kidney and then secreted into the blood to play a role. GPX4 is one of the strongest antioxidant enzymes in the human body It can convert reduced GSH in the cell into oxidized glutathione (GSSG) and at the same time convert the toxic lipid hydrogen peroxide in the cell into non-toxic ester alcohol and regulate intracellular redox balance and protect cell membrane structure and function (15). Erastin inhibits GSH production by inhibiting System Xc^-^ and preventing cystine uptake. When GSH is depleted, it will cause GPX4 inactivation and cause ferroptosis (16). Hao et al. (17) discovered that silencing cysteine ​dioxygenase 1 (CDO1) can inhibit Erastin-induced ferroptosis of gastric cancer cells. Inhibition of CDO1 can restore cell GSH levels, prevent the production of ROS, and reduce malondialdehyde, which is one of the final products of lipid peroxides. Hayano et al. (18) found in the study of the mechanism of ferroptosis that knocking out the gene producing cysteamide-transport RNA synthetase significantly inhibited the ferroptosis caused by Erastin.

### Iron Metabolism

Iron is an indispensable and most abundant trace element in the body, and it participates in many important physiological and biochemical functions in the body. Iron is the main raw material for the synthesis of hemoglobin and myoglobin. It not only participates in the biosynthesis of DNA and ATP but is also an important electron transport chain in mitochondria and a cofactor of metalloproteinases (19). Under normal conditions, the body maintains the homeostasis of iron through food sources of iron and the “iron cycle” (a process in which aging red blood cells release iron ions under the action of heme oxygenase, and macrophages re-engulf and recycle iron). The hepcidin synthesized and secreted by the liver directly regulates the level of serum iron, while the regulation of iron homeostasis in the body’s cells is mainly played by the iron responsive element, the hepcidin system (20). The trivalent iron ion (Fe^3+^) in the peripheral circulation combines with transferrin to form a complex and then binds to the transferrin receptor on the cell membrane and enters the endosomes in the cell. At this time, Fe^3+^ is reduced to divalent iron ions (Fe^2+^) through the Steap3 (six- transmembrane epithelial antigen of the prostate), and then under the mediation of Transferrin Receptor-1(TFR-1), Fe^2+^ is released from the endosome to the cytoplasm, and part of it is stored in unstable iron tanks. The excess iron is stored in the iron storage protein complex composed of ferritin light chain polypeptide and ferritin heavy chain polypeptide 1, and the remaining part of Fe^2+^ will be oxidized to Fe^3+^ and transferred out of cells to participate in iron renewal in the body cycle (21). Increased iron intake or decreased iron output can enhance the sensitivity of cancer cells to oxidative damage and ferroptosis. Fe^2+^ level in cells is a key factor for lipid peroxidation and induction of ferroptosis, and TF-mediated iron uptake or iron autophagy can increase its level (3). Iron autophagy is the selective autophagy of ferritin, which increases the sensitivity to ferroptosis by controlling the available iron (22). Nuclear receptor coactivator 4 (NCOA4) is a specific transport receptor for iron autophagy, which can transport ferritin to autophagosomes for lysosomal degradation and release of free iron (23).

### Lipid Metabolism and ROS

Lipids are important regulators of cell death. In mammals, both apoptosis and non-apoptotic pathways can be induced, regulated or inhibited by different lipid signals (24). Unlike other types of cell death, ferroptosis does not require protein effectors like pore-forming proteins. Lipid oxidative stress and the membrane damage caused by it are the key to ferroptosis, especially polyunsaturated fatty acids (PUFA), which forms lipid peroxides and induces ferroptosis (25). PUFA participates in the lipid bilayer structure that composes the cell membrane, and it is an important target of lipid peroxidation in the ferroptosis process of the cell membrane (2).

ROS are produced by normal physiological processes and play an important role in cell signal transduction and tissue homeostasis. However, excessive reactive oxygen free radicals produce unfavorable modifications to cell components, such as lipid, protein and DNA damage (26). Biological cell membranes or organelle membranes have lots of PUFAs and they are particularly vulnerable to ROS damage, which is called “lipid peroxidation” (27). Lipid peroxidation induced by ROS plays an important role in cell death. Lipid peroxidation directly damages phospholipids and can also be used as a cell death signal to induce programmed cell death. Accompanied by the accumulation of ROS, ferroptosis is induced by erastin and RSL3 (a selective ferroptosis inducer). It was found that ferrostatin-1 (Fer-1) and lipoxstatin-1 (Lip-1) can prevent erastin-induced accumulation of ROS, which also verifies the important role of ROS accumulation in promoting ferroptosis (28).

## Ferroptosis and Tumors

### Glioma

Gliomas, especially glioblastoma, are the most common malignant tumors in the brain. The incidence rate of glioma is about 6/100 000 and is steadily increasing (29). Through research on ferroptosis, it is found that some transcription factors play an important role in the proliferation, migration and malignant transformation of glioma, and their mechanism of action is closely related to ferroptosis (30).

Chen et al. (31) found that when the expression of ATF4 increases, activated ATF4 will increase the expression of Xc^-^. The inhibition of Xc^-^ can promote the occurrence of glioma cell ferroptosis. and studies have shown that down-regulating the expression of ATF4 can Inhibit the activity of Xc^-^, thereby enhancing the sensitivity of nerve tumor cells to ferroptosis, so that the proliferation and production of tumors and their vasculature can be controlled. Therefore, ATF4 may be an effective target for inhibiting tumor cell proliferation and tumor blood vessel growth. Fan et al. (32) found that the overexpression of Nrf2 and the knockout of Keap1 can promote the proliferation and migration of glioma tumor cells; the mechanism may be through Up-regulating the activity of Xc^-^ changes the tumor microenvironment and inhibits the ferroptosis of tumor cells. Down-regulating the expression of Nrf2 in glioma cells can promote ferroptosis of tumor cells. The mechanism may be through inhibiting the activity of Xc^-^, reducing the secretion of glutamate, increasing the production of intracellular lipid ROS, and promoting ferroptosis. It was also found that Nrf2 controlled glutathione synthesis and plays a central role in IDH1 mutated glioma cell physiology (33). At the same time, down-regulating the expression of Nrf2 can increase the sensitivity of glioma cells to inducers of ferroptosis (such as erastin, RSL3).

Pseudolaricacid B (PAB) has been shown to inhibit the growth of glioma cells in animal and cell experiments (34). Because it can increase the content of ferrous iron in cells, and intracellular iron can regulate the expression of NOX4, thereby increasing intracellular hydrogen peroxide and the formation of lipid ROS; PAB can also inhibit the function of SLC7A11 by activating P53, reducing the content of GPX4 in the cell, lead to accumulation of lipid ROS in the cell, and ultimately lead to ferroptosis of glioma cells (35).

Temozolomide (TMZ) is an effective drug for the treatment of high-grade gliomas, which can delay the survival of patients (36). Sehm et al. (37) found that TMZ combined with ferroptosis inducers can enhance the therapeutic effect. Experiments have found that when TMZ is combined with erastin, it can increase its anti-cancer effect. Up-regulation of Xc^-^ expression will make tumor cells more effective. The therapeutic effect of TMZ combined with erastin is more sensitive. Gao et al. (38) found that ibuprofen can down-regulate the expression of GPX4 and Xc^-^ in glioblastoma, and the decrease in the expression of GPX4 and Xc^-^ is related to the down-regulation of Nrf2 expression. Nrf2 can regulate the expression of GPX4 and Xc^-^ in glioblastoma. The expression of ibuprofen may inhibit the activity of GPX4 and Xc^-^ by down-regulating the expression of Nrf2, which increases the production of lipid ROS in tumor cells, which in turn promotes the occurrence of ferroptosis in glioblastoma.

### Lung Cancer

Lung cancer is one of the most common malignant tumors in the world. In 2018, there are an estimated 2.1 million new cases and nearly 1.7 million deaths, while the incidence of lung cancer and mortality ranks first among all tumors in China (39). Epidemiological and laboratory studies have confirmed that iron overload is related to the occurrence and development of lung cancer, and there is a significant positive correlation between high iron intake and lung cancer risk. Data from a clinical trial showed that the serum iron, ferritin, and total iron binding capacity of lung cancer patients were significantly higher than those of healthy controls. The higher the serum iron concentration, the greater the risk of lung cancer (40). A study in Taiwan enrolled 309,443 people from 2018 to 2009. The median follow-up time of non-tumor population was 7.07 years, of which 8,060 cases were diagnosed with tumors and 3,066 cases of Death due to tumor, high serum iron (>120 μg/dL) increases the risk of morbidity and death of malignant tumors and is positively correlated with tumor morbidity and mortality (41).

In recent years, some researchers have discovered that ferroptosis is suppressed in lung cancer cells. Ji et al. (42) found that lung cancer cells can directly target GSH by up-regulating System Xc^-^ to increase cellular antioxidant capacity. Lai et al. (43) found that lung cancer cells inhibit ferroptosis by directly upregulating the expression of GPX4. Serine threonine tyrosine kinase 1 (STYK1) is highly expressed in NSCLC cells, which in turn promotes the expression of GPX4 and promotes the Proliferation of lung cancer cells, which attenuates a variety of mitochondrial abnormalities caused by ferroptosis, leading to suppression of ferroptosis in NSCLC. FSP1 is an ferroptosis inhibitor independent of the classical GPX4 signaling pathway (44). When the GPX4 gene of lung cancer cells is deleted, FSP1 would be modified by myristoylation, and CoQ10 is reduced by NAD (P) h to produce radial trapping antioxidants (RTA) to prevent lipid peroxidation and inhibit ferroptosis (45). The higher the expression level of FSP1, the greater the ferroptosis resistance of lung cancer cells. FSP1 inhibitor (ifsp1) can reverse the ferroptosis resistance caused by FSP1, increasing the sensitivity of lung cancer cells to ferroptosis and promoting ferroptosis of lung cancer cells (46).

Among the drugs for the treatment of lung cancer, some have been proved to induce ferroptosis. Cisplatin (DDP) promotes lipid peroxidation, increases MDA, ROS, promotes the expression of HO-1 and NQO-1, and induces ferroptosis of lung cancer cells, and this process can be inhibited by Fer-1 (47). The activation of the Nrf2/Xc^-^ pathway is one of the main mechanisms of NSCLC cell resistance to cisplatin. Erastin and sorafenib inhibit the expression of Nrf2 downstream target gene Xc^-^ deplete GSH, induce ferroptosis, reduce cell viability, and enhance the sensitivity of NSCLC cells to cisplatin (48). On the contrary, overexpression of SLC7A11 enhances the resistance of lung cancer cells to cisplatin (49).

Ferroptosis may play a role in lung cancer radiotherapy. After radiotherapy, tumor cells showed typical ferroptosis morphological changes—mitochondria were concentrated, membrane density increased, and mitochondrial ridges decreased (50). Radiotherapy can generate a large amount of ROS and up-regulate the expression of key enzymes to promote lipid peroxidation and eventually lead to ferroptosis (51).

### Hepatocellular Carcinoma

Hepatocellular carcinoma (HCC) is one of the malignant tumors that cause a serious global burden of disease, ranking sixth among the most common cancers and second among cancer-related deaths (52). In recent years, many studies have confirmed that HCC is related to ferroptosis (53). Ferroptosis is mainly regulated by system Xc^-^ and GPX4 and affecting the activity of system Xc^-^ or GPX4 can induce ferroptosis of liver cancer cells (54). p53 is a tumor suppressor gene that affects the occurrence and development of HCC by regulating ferroptosis (55). Xie et al. found that p53 down-regulates the transcription of SLC7A11 and affects the activity of system Xc^-^, thereby inducing ferroptosis of liver cancer cells (56). Glutaminase-2 is a key enzyme for the conversion of glutamine to glutamate and regulates GSH synthesis. p53 up-regulates glutaminase 2 transcription, and its overexpression inhibits HCC tumor cell growth and colony formation (57).

Sorafenib, as a first-line drug for advanced HCC, has been proven to induce ferroptosis in HCC cells. Sorafenib can inhibit the expression of SLC7A11 and activate the downstream ferroptosis pathway with its own RAF kinase inhibitor effect (58). Sun et al. (8) found that liver cancer cells under oxidative stress activate their own P62-Keap1-NRF2 signaling pathway to up-regulate target genes involved in iron and ROS metabolism downstream of NRF2, such as quinone oxido-reductase 1 (NQO1), heme oxygenase 1 (HO-1) and ferritin heavy chain-1 (FTH1), thereby enhancing Sorafenib’s ferroptosis resistance (59). In addition, a psychotropic drug, haloperidol, was reported to have a sensitizing effect on sorafenib through the ferroptosis route (60).

### Osteosarcoma

Osteosarcoma is the most common primary bone malignant tumor, which is common in young people. The 5-year survival rate is 60% ~ 70%, and the mortality caused by lung metastasis is 30% ~ 40% (61).

WU et al. (34) reported the high expression of TFR1 in osteosarcoma and further confirmed that the high expression of TFR1 is significantly related to the histological grade, stage and distant metastasis of the tumor (62). TFR1 is the main protein for iron absorption, which is very important in ferroptosis. Liu et al. (63)found that promoting ferroptosis of osteosarcoma cells can enhance the sensitivity of target cells to cisplatin. Further research found that the resistance of osteosarcoma cells to cisplatin was also enhanced after treatment of non-resistant strains with ferroptosis inhibitors (64).

### Ovarian Cancer

Ovarian cancer was the seventh most common women cancer in the world, with around 240,000 new cases each year. Ovarian cancer is the second most common malignancy in women over the age of 40, particularly in developed countries (65). It also has many connections with ferroptosis. Basuli et al. (66) found that increased intracellular iron levels are related to the occurrence of ovarian cancer. Compared with normal ovarian tissue, high-grade serous ovarian cancer tissues have decreased FPN, increased TFR1 and TF, and increased iron levels. The genetic model of the initiating cells of ovarian cancer also shows that the iron outflow pump is reduced, and the expression of iron transport-related proteins is up-regulated (67).

Many studies have confirmed that increased intracellular glutathione levels and high expression of related metabolic enzymes are closely related to the drug resistance of ovarian cancer (68). Liu et al. (69) established an Erastin-resistant cell line and found that the cell line can still maintain the content of glutathione, suggesting that there are other ways to synthesize cystine in the cell. Verschoor et al. (70) used Xc^-^ system inhibitors and transsulfide pathway inhibitors to treat two ovarian cancer cell models, and found that intracellular glutathione levels were significantly reduced after the transsulfuration pathway was inhibited, indicating that the transsulfuration pathway has an effect on ovarian cancer cells. The synthesis of glutathione is very important. Chakraborty et al. (71) found that in a small number of ovarian cancer cell lines, the expression of CBS in the transsulfide pathway was increased, and CBS gene silencing could inhibit cell migration and invasion of ovarian cancer cells.

Artemisinin is a classic antimalarial drug. The cellular response of tumor cells to artemisinin and its derivatives involves ferroptosis, apoptosis, necrosis and other cell death methods (72). After the treatment of ovarian cancer cells with artemisinate, a large amount of ROS is produced in the cells, and the proliferation is reduced, which shows that artemisinin may play a role in ovarian cancer therapy (73).

## Conclusion

Ferroptosis is an important form of regulatory necrosis, which is different from other cell necrosis and apoptosis in morphology, biochemistry and genetics. The mechanism of ferroptosis is closely related to cell metabolism, involving a variety of key molecules and signal pathways, and regulating the synthesis or decomposition of these key molecules and the signal pathways involved will change the sensitivity of cells to ferroptosis. Reasonable induction or inhibition of ferroptosis will improve the treatment of a variety of diseases, especially cancer-associated malignancies. However, many problems remain to be solved in anti-tumor treatments that target ferroptosis. Firstly, several studies have shown that inhibiting the expression of GPX4 gene can effectively kill tumor cells through ferroptosis. Furthermore, GPX4 is a candidate prognostic biomarker for many cancers (74), so it is necessary to further clarify whether GPX4 is an oncogene. Secondly, immunotherapy is a relatively new anti-tumor treatment, and its relationship with ferroptosis is in the preliminary exploration stage. The relevant mechanism has not yet been fully clarified, and further research is needed. Thirdly, Although some scholars have proposed initial ideas to target SLC7A11 for cancer treatment, such as directly inhibiting the activity of SLC7A11 transport protein, or starting from the metabolic vulnerability of cancer and SLC7A11. However, further research and marketing of drugs targeting SLC7A11 is still necessary, and more studies are needed to determine the safety and efficacy of p53-related drugs (75). In addition, whether the nanomedicine designed with ferroptosis as the target has obvious anti-tumor effects in the human body (76) should be further confirmed by clinical experiments. In short, opportunities and challenges coexist. Ferroptosis is a promising target for anti-tumor therapy and its clinical application will surely bring good news to cancer patients.

## Author Contributions

CY: Conceptualization, writing - original draft, writing - review and editing. ZF: Formal analysis, investigation, writing - review and editing. SH: Investigation, Resources, Writing - review and editing. CL, Supervision, writing - review and editing. YX: Grammar curation, investigation. SL: Supervision, editing. ZF and SH are co-first authors. All authors contributed to the article and approved the submitted version.

## Conflict of Interest

The authors declare that the research was conducted in the absence of any commercial or financial relationships that could be construed as a potential conflict of interest.

## Publisher’s Note

All claims expressed in this article are solely those of the authors and do not necessarily represent those of their affiliated organizations, or those of the publisher, the editors and the reviewers. Any product that may be evaluated in this article, or claim that may be made by its manufacturer, is not guaranteed or endorsed by the publisher.
